# The HBV variant CpF97L supports the secretion of pgRNA-containing virions at a level much greater than WT HBV

**DOI:** 10.1128/jvi.00100-25

**Published:** 2025-04-15

**Authors:** Abena Adomah Kissi-Twum, Karolyn Pionek, Daniel D. Loeb

**Affiliations:** 1 McArdle Laboratory for Cancer Research, School of Medicine and Public Health, University of Wisconsin-Madison 5228, Madison, Wisconsin, USA; 2 Microbiology Doctoral Training Program, University of Wisconsin-Madison 5228, Madison, Wisconsin, USA; Wake Forest University School of Medicine, Winston-Salem, North Carolina, USA

**Keywords:** HBV, genome replication, pgRNA virion, CpF97L

## Abstract

Viruses in the *Hepadnaviridae* family, including hepatitis B virus (HBV), replicate their double-stranded DNA (dsDNA) genomes through reverse transcription of an RNA intermediate, the pregenomic RNA (pgRNA), in the viral capsid within an infected cell. In the cell, capsids containing pgRNA, single-stranded DNA (ssDNA), and dsDNA are present. However, capsids containing dsDNA (referred to as mature genomes) are preferentially secreted in virions while only small amounts of capsids with pgRNA and ssDNA (referred to as immature genomes) are enveloped and secreted. The naturally occurring HBV core protein variant, CpF97L, is an exception; HBV CpF97L secretes high levels of ssDNA-containing virions in addition to dsDNA virions. We asked whether HBV CpF97L is capable of secreting pgRNA-containing virions as well. We found that HBV CpF97L secretes high levels of pgRNA-containing virions compared to wild-type (WT) HBV when reverse transcription was inhibited by entecavir or by the Y63F change in P protein. We detected pgRNA virions in Huh7 and HepG2 cell lines, indicating that RNA virion secretion was independent of the cell line used in virion propagation. More importantly, pgRNA virions were detected when dsDNA virions were synthesized as well. Our findings suggest that the capsids of CpF97L are constitutively matured, allowing for virions with immature genomes (ssDNA and pgRNA) to be secreted in addition to dsDNA virions.

**IMPORTANCE:**

Finding a cure for hepatitis B is critical, as over 250 million people live with a hepatitis B virus (HBV) infection. HBV replicates through a series of nascent RNA and DNA intermediates in capsids, resulting in the secretion of a DNA virion to propagate the infection. HBV infections have been managed with nucleos(t)ide analogs (NAs), which terminate DNA synthesis during replication. During NA treatment, DNA levels plummet, RNA-containing capsids accumulate in infected cells and are secreted, albeit inefficiently, as virions. RNA virions in serum have therefore been proposed to be used as an indicator for covalently closed circular DNA (cccDNA) (HBV’s minichromosome in hepatocytes) to determine patients who can be withdrawn from NAs without virological rebound. However, it is unknown if RNA virions are efficiently secreted by the frequent HBV variants that secrete high levels of ssDNA-containing virions, as these will lead to an erroneous overestimate of the cccDNA reservoir; hence, the need for our study.

## INTRODUCTION

Chronic hepatitis B virus (HBV) infection remains a global public health threat with over 254 million people living with the disease and 1.2 million people newly infected yearly (1). The WHO estimates that 1.1 million people die each year from chronic hepatitis B (CHB) due to cirrhosis and cancer of the liver. HBV is a member of the *Hepadnaviridae* family; these are enveloped viruses with a partially double-stranded DNA (dsDNA) that primarily infect the liver. This family of viruses synthesizes their genomes in the viral capsid through a series of RNA and DNA intermediates generated via reverse transcription (2, 3).

To initiate an infection, the HBV virion enters a cell through the sodium taurocholate co-transporting polypeptide (NTCP) receptor on a hepatocyte (4). The capsid of the virion is deposited into the cytoplasm of the infected cell from where it traffics to the nucleus to deposit its dsDNA genome. In the nucleus, the partially dsDNA genome is converted by the cellular DNA repair machinery to form the covalently closed circular DNA (cccDNA) (5). The cccDNA persists (6) in the nucleus and serves as the template for transcription of HBV RNAs by the host RNA polymerase II enzyme (7).

One of the RNAs transcribed from the cccDNA is the pregenomic RNA (pgRNA) (2). The pgRNA is multifunctional; it is the mRNA for the viral core (Cp) and polymerase (P) proteins, and the template for reverse transcription (8, 9). Dimers of Cp form the viral capsid and encapsidate pgRNA and polymerase (10–12). In the capsid, the HBV polymerase reverse transcribes pgRNA into minus-strand DNA (ssDNA) with concomitant degradation of the pgRNA by the RNase H domain of the viral polymerase (2). The minus-strand ssDNA molecule serves as the template for synthesis of plus-strand DNA to generate dsDNA. Capsids containing dsDNA genomes are preferentially secreted as enveloped virions while capsids containing pgRNA or ssDNA remain in the cytoplasm to mature into dsDNA. Although dsDNA-containing capsids are preferentially secreted as virions, pgRNA- and ssDNA-containing virions are synthesized at roughly 0.1% of dsDNA-containing virions (13).

Preferential secretion of dsDNA-containing virions is a fundamental characteristic of the *Hepadnaviridae* family as it has been observed with almost all family members (14). To explain this phenomenon, the capsid maturation hypothesis posits that a maturation signal appears on capsids upon the synthesis of the dsDNA genome and allows for the interaction of the capsid with envelope proteins and subsequent virion morphogenesis (2). Capsids containing ssDNA and pgRNA genomes lack this signal and thus are not enveloped nor secreted as virions at a high level. The single-strand blocking model (15) is a permutation of the capsid maturation hypothesis; however, in this model, the single-stranded genomes (pgRNA and ssDNA) block capsid envelopment and virion morphogenesis. The single-strand blocking model is consistent with the observation of dsDNA-containing and empty capsids secretion in virions (15, 16).

A frequently occurring Cp mutation identified in patients (17–19) has been shown to result in the secretion of ssDNA-containing virions at levels greater than dsDNA-containing virions in cell culture-based assays (20). A single amino acid change of Phenylalanine (F) or Isoleucine to Leucine (L) at codon 97 of Cp (CpF97L or CpI97L) was identified as sufficient for the secretion of ssDNA-containing, in addition to dsDNA-containing virions. In our work, we found that the HBV-CpF97L additionally secretes high levels of RNA-containing virions.

## RESULTS

The naturally occurring HBV Cp variant, CpF97L, has been shown to secrete ssDNA virions in addition to dsDNA virions (20). We asked whether CpF97L also secreted pgRNA-containing virions and, if so, to what level. We used three different approaches to answer this question. In all approaches, HBV replication was initiated via transfection of cell cultures, and intracellular HBV replication and virion production were evaluated. All data presented represent at least three experiments, each consisting of two independently transfected plates.

### When DNA synthesis was inhibited, CpF97L secreted high levels of pgRNA-containing virions

We inhibited DNA synthesis using two methods: a pharmacological method using entecavir (ETV), a nucleoside analog of deoxyguanosine, and a genetic method by introducing the Y63F mutation into the HBV polymerase. The HBV plasmids WT^RT-^ and CpF97L^RT-^ contained the P^Y63F^ mutation to prevent the initiation of synthesis of minus-strand DNA (21). Huh7 cell cultures were transfected with plasmids expressing WT^RT-^, CpF97L^RT-^, wild type HBV (WT), and CpF97L. The cultures expressing WT or CpF97L were treated with ETV or vehicle (DMSO). The cells and media were then harvested and analyzed.

**Fig 1:**
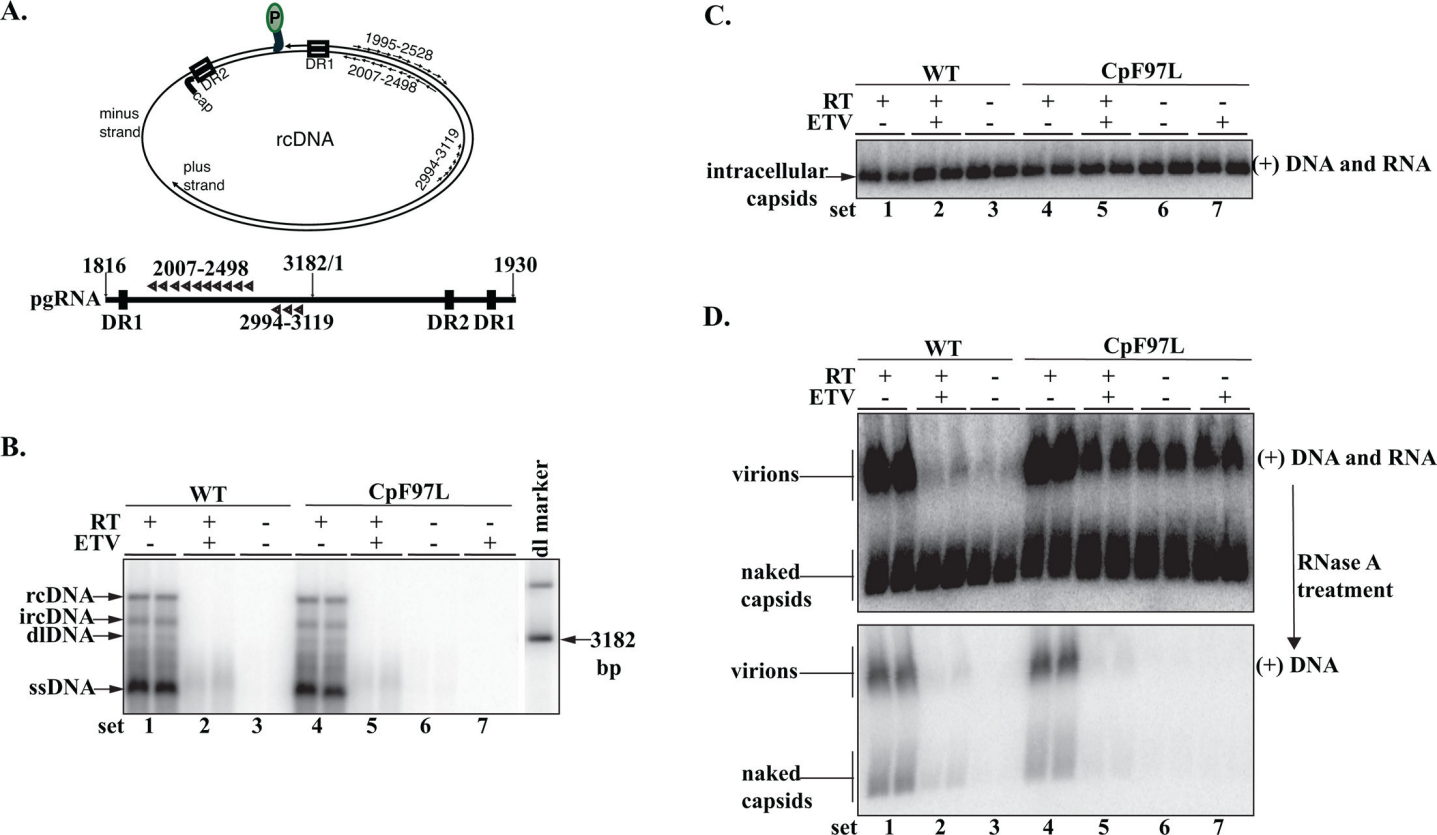
HBV CpF97L secretes RNA virions efficiently when reverse transcription is inhibited genetically or pharmacologically. (**A**) The relative positions of oligonucleotide probes on the rcDNA and pgRNA molecules. The 5′-end of plus probe: 2007- to 2498-; the 3′-end of minus probe: 1995+ to 2528+; and the major splice intron probe: 2994- to 3119-. Only probes that detect the plus strand are indicated on the pgRNA (2007- to 2498- and 2994- to 3119-). (**B**) Southern blotting of intracellular capsid DNA probed for the minus strand. Each lane in a set represents DNA extracted from 2/5^th^ of a 60 mm dish of transfected Huh7 cells. RT-, Y63F mutation in P gene; ETV+, treatment with 1 uM of ETV. The dl marker comigrates with the duplex linear DNA-dlDNA. We detected a band between rcDNA and dlDNA; this is rcDNA with an incompletely elongated plus strand (ircDNA) as previously described (23). (**C**) Particle blot analysis of intracellular capsids probed for the plus-strand nucleic acid (pgRNA and dsDNA). Each lane in a set represents 1/50^th^ of the cytoplasmic lysate from a 60 mm dish of transfected cells. (**D**) Particle blot analysis of media probed for the 5′-end plus-strand nucleic acid (pgRNA and dsDNA, top panel). The membrane was treated with RNase A (bottom panel) and re-probed for the 5′-end of plus strand. An aliquot of 1/3^rd^ (4 mL out of 12 mL) of the pooled media harvested from the 60 mm plate was loaded in each lane of a set.

First, we found, as expected, that transfected cells treated with ETV in addition to WT^RT-^ and CpF97L^RT-^ transfected cells did not synthesize detectable levels of DNA by performing Southern blotting on DNA extracted from intracellular capsids. As expected, DNA synthesis was significantly inhibited in intracellular capsids of WT and CpF97L cell cultures treated with 1 µM ETV (Fig. 1B, sets 2 and 5), as observed previously (22). Low levels of ssDNA were observed in the ETV-treated samples. As expected, WT^RT-^ and CpF97L^RT-^ cultures did not synthesize detectable levels of DNA (Fig. 1B, sets 3, 6, and 7). As a comparison, WT and CpF97L, which do not contain the Y63F substitution in P nor were treated with ETV, synthesized robust levels of HBV capsid DNA (Fig. 1B, sets 1 and 4).

Next, we found, as expected, that in the cells where DNA was not detected, RNA-containing capsids accumulated intracellularly. We demonstrated this via particle gel blotting using plus-strand hybridization probes that detected the 5′-end of pgRNA (and dsDNA on the WT and CpF97L comparisons, Fig. 1A). We detected HBV nucleic acids of plus polarity in the intracellular capsids isolated from WT^RT-^ and CpF97L^RT-^ (Fig. 1C, sets 3 and 6) in addition to WT and CpF97L treated with ETV (Fig. 1C, sets 2 and 5). As expected, neither the ETV treatment nor the Y63F mutation prevented the packaging of RNA into capsids (Fig. 1C, sets 2, 3, 5–7).

We then determined if the intracellular capsids containing RNA were secreted in virions. Media harvested from these cultures were analyzed by native agarose particle blotting and probed with the same set of plus-strand-detecting oligonucleotides described above (5′-end plus-strand probe set, Fig. 1A). Robust levels of RNA-containing virions were detected in CpF97L^RT-^ samples. pgRNA-containing virions were secreted from cultures expressing CpF97L^RT-^ and cultures expressing CpF97L treated with ETV (Fig. 1D, sets 5–7) at levels comparable to those observed for CpF97L or WT (Fig. 1D, sets 1 and 4). This was in sharp contrast to the level of virions secreted for the ETV-treated WT or WT^RT-^ (Fig. 1D, sets 2 and 3). To corroborate that the nucleic acids detected for the CpF97L^RT-^ and ETV-treated CpF97L cultures were RNA, we treated the membrane with RNase A to degrade RNA molecules on the membrane. RNase A treatment followed by reprobing of the membrane led to the loss of most of the HBV plus-strand nucleic acid in the ETV-treated samples (Fig. 1D, sets 2, 5, and 7), indicating the nucleic acid content of the capsids in the ETV-treated samples and the samples with the Y63F mutation in P was predominantly, if not totally, RNA.

To summarize the findings presented in Fig. 1, using ETV or the genetic change P^Y63F^ in WT^RT-^ and CpF97L^RT-^, we demonstrated that virions containing pgRNA were made whenever the CpF97L mutation was present in the expression plasmid. This contrasts with WT, which secreted RNA virions at significantly lower levels under similar conditions.

### HBV CpF97L^RT-^ secreted RNA virions at levels comparable to dsDNA virions while WT^RT-^ does not

For our subsequent analysis, we used WT^RT-^ and CpF97L^RT-^ plasmids only to generate RNA capsids and virions. We proceeded with these plasmids because DNA was not detected by Southern blotting analysis (Fig. 1B, sets 3 and 6 compared to low levels of DNA in ETV-treated samples, sets 2 and 5) and on RNase A treatment of particle gel membranes (Fig. 1D, bottom panel).

**Fig 2:**
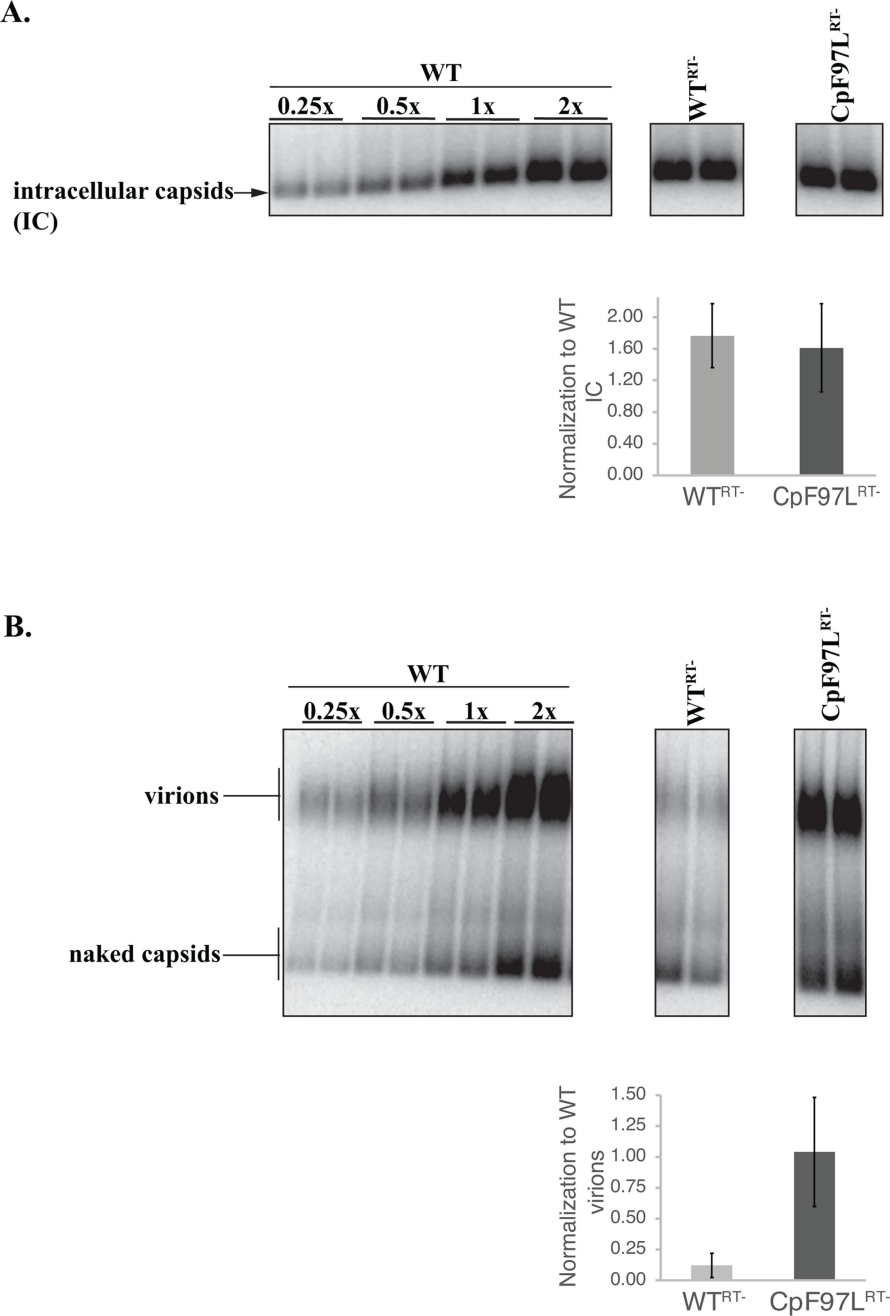
HBV CpF97L^RT-^ secretes RNA virions at levels comparable to dsDNA while WT HBV does not. Particle blot analysis of intracellular capsids (IC) (**A**) and media (**B**) harvested from days 3, 5, and 7 of transfected Huh7 cells probed for plus-strand nucleic acids (dsDNA and pgRNA). The WT standard curve ranged from 0.25x to 2x volumes. An aliquot of 1/100^th^ of the cytoplasmic lysate (**A**) and 1/12^th^ of the media (**B**) collected from each transfected plate was analyzed. Data in bar charts (mean ± standard deviation) represent the average of three experiments, each consisting of two independently transfected plates.

We asked how much pgRNA was detected in intracellular capsids and how much was secreted in virions in WT^RT-^ and CpF97L^RT-^. To quantify pgRNA in intracellular capsids (IC) and virions, we generated standard curves from WT cultures (Fig. 2). The relative expression of WT^RT-^ and CpF97L^RT-^ samples was determined from the WT standard curve.

We demonstrated, as expected, that cells expressing WT^RT-^ and CpF97L^RT-^ accumulated similar amounts of intracellular RNA-containing capsids (Fig. 2A). We demonstrated this via particle gel blotting using hybridization probes that detected the 5′-end of pgRNA.

We then asked how much pgRNA-containing virions were secreted by WT^RT-^ and CpF97L^RT-^ cultures, respectively. The media harvested from these cultures were analyzed by particle blotting and probed with the same set of plus-strand-detecting oligonucleotides described above (5′-end plus-strand probe set). Robust levels of RNA-containing virions were detected in CpF97L^RT-^ samples as already established (Fig. 2B), whereas low levels of RNA-containing virions were observed in WT^RT-^ samples. In fact, compared to the WT standard curve, CpF97L^RT-^ secreted 1.04 ± 0.44 pgRNA-containing virions (Fig. 2B) comparable to the virion levels observed in WT which synthesizes dsDNA virions. CpF97L^RT-^ secreted about eightfold more pgRNA-containing virions than WT^RT-^, which secreted 0.12 ± 0.10 virions in media (Fig. 2C).

In summary, the HBV variant, CpF97L, secreted RNA virions at levels comparable to dsDNA virions when DNA synthesis was inhibited.

### RNA virion secretion was observed in Huh7 and HepG2 cell lines

**Fig 3:**
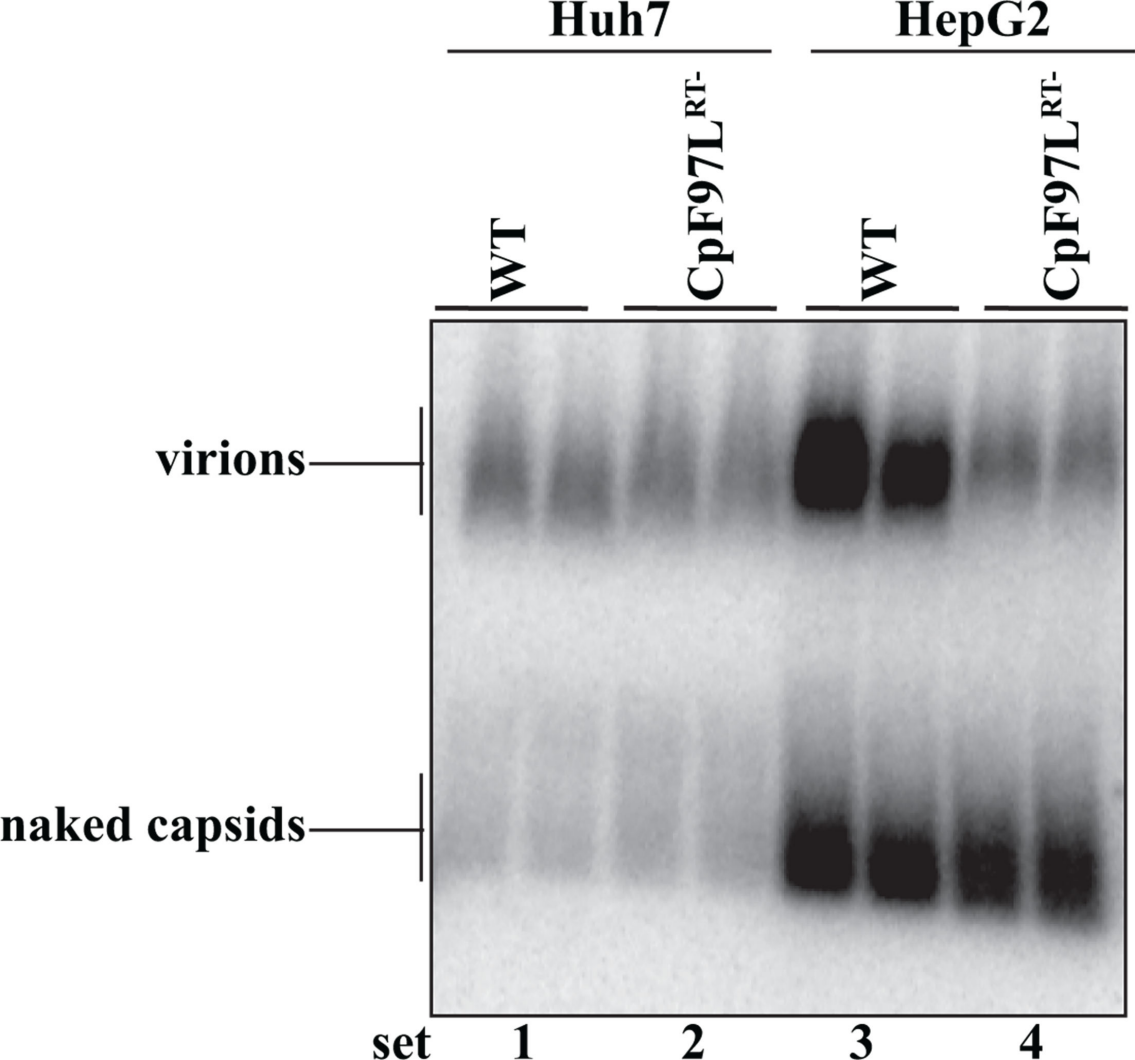
RNA virion secretion is a property of CpF97L and is independent of the cell line used in transfection. Particle blotting analysis with the resulting membranes probed for the 5′-end plus-strand nucleic acid (nts 2007- to 2498-). Each lane in a set contains 1/3^rd^ of the medium from each transfected plate.

The analysis up to this point used the Huh7 cell line (Fig. 1 and 2). We wanted to determine if the secretion of virions containing RNA by CpF97L was solely due to an idiosyncrasy of the Huh7 cell line. To this end, we transfected the HepG2 cell line with CpF97L^RT-^ and determined if RNA virions were secreted. As a comparison, WT virions were included in this analysis. Virions were analyzed by particle gel blotting as was previously carried out in Fig. 1 and 2. The results indicated that RNA-containing virions were made by HepG2 cells (Fig. 3, set 4), as has been observed in Huh7 cells, albeit to a lesser extent than WT.

### The kinetics of secretion of RNA-containing virions and dsDNA-containing virions are similar

**Fig 4:**
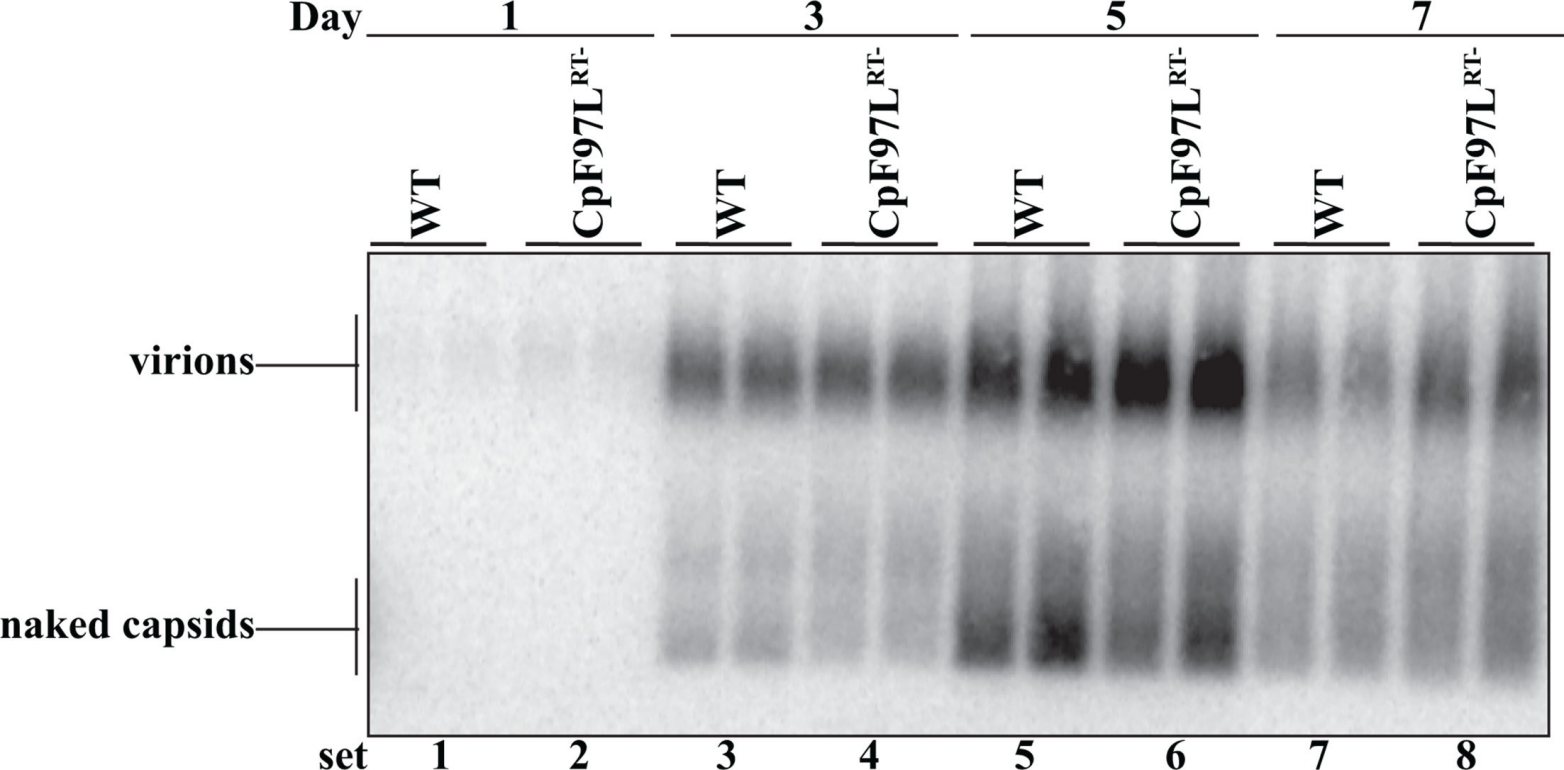
The kinetics of secretion of DNA virions and RNA virions are similar. Particle gel blotting of medium from cells transfected with WT and CpF97L^RT-^ probed for the 5′-end of plus strand. Each lane represents the total media harvested (4 mL of media) at each of the denoted time points.

Collectively inside cells replicating HBV, capsids contain a continuum of immature and mature genomes ranging from newly encapsidated pgRNA to fully double-stranded DNA and everything in between. However, WT HBV preferentially secretes as virions only a subset of capsids, those that contain dsDNA. Given that CpF97L behaves differently and can synthesize RNA-containing virions, we wanted to determine whether the kinetics of secretion of RNA-containing virions were similar or not to the kinetics of secretion of dsDNA-containing virions. To this end, we transfected WT or CpF97L^RT-^ plasmids into Huh7 cells. We harvested media from the cultures 24 hours after transfection and every 48 hours subsequently until day 7. Virion levels were determined by particle gel blotting (Fig. 4). The pattern of accumulation of virions for WT (which secretes predominantly dsDNA virions) and CpF97L^RT-^ (which secretes only RNA virions) looked similar, if not indistinguishable (Fig. 4); little to no virions were detected on day 1 (Fig. 4, sets 1 and 2), significant levels at day 3 (Fig. 4, sets 3 and 4), which peaked at day 5 (Fig. 4, sets 5 and 6), followed by a decline at day 7 (Fig. 4, sets 7 and 8). This analysis suggested that the CpF97L mutation did not alter the kinetics of secretion of virions even though only RNA-containing virions were made in these cultures.

### The RNA in CpF97L^RT-^ virions is ~3,500 nt in length

Our analysis thus far has detected RNA virions by particle gel blotting, a method useful for determining the presence of nucleic acids in virions but not the size or heterogeneity of their nucleic acid content. To this end, we purified virions via immunoprecipitation (IP) with an HBsAg antibody and extracted RNA, then performed Northern blotting. We also extracted RNA from intracellular capsids from the same cultures and included them in the Northern blotting. Additionally, we included an *in vitro* transcribed pgRNA (IVT pgRNA) as a size marker (24). Lastly, total poly A+ RNA was isolated from Huh7 cells transfected with WT^RT-^ and included in the Northern blotting. We analyzed the Northern blots with a probe set that detected the 5′-end of plus strand (pgRNA, nt 2007- to 2498-).

**Fig 5:**
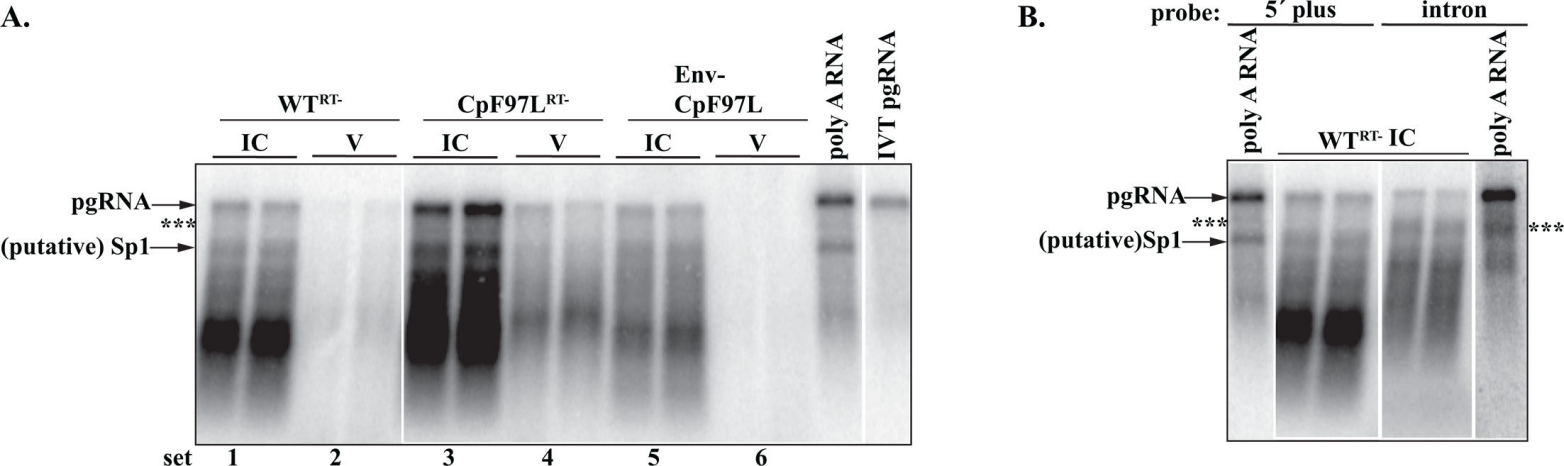
Full-length pgRNA is detected in RNA virions. (**A**) Northern blotting of intracellular capsids (IC) and virion (V) RNA. IVT pgRNA, *in vitro* transcribed pgRNA; poly A RNA, total poly A+ RNA extracted from WT^RT-^. IC samples represent the RNA from 1/10^th^ of the cultured plate; V samples represent the RNA from 1/3^rd^ of the total medium harvested from the transfection. The membrane was probed for the 5′-end of plus strand (ayw nt 2007- to 2498-). (B) The hybridization probes were removed from the membrane in panel (A), and the membrane was re-probed for the major splice intron on the plus strand (oligonucleotides 2994- to 3119-). Only the poly A+ RNA and WT^RT-^ IC are shown for simplicity; CpF97L^RT-^ IC and V samples showed identical profiles to WT^RT-^ IC samples shown here. Representative data in Figure 5 and 6 were analyzed together on the same membrane.

The topmost band in the intracellular capsid samples (IC) for WT^RT-^ and CpF97L^RT-^ (Fig. 5A, sets 1 and 3) co-migrated with the *in vitro* transcribed pgRNA transcript and poly A RNA, consistent with it being pgRNA. A similar, if not identical, profile was observed in secreted RNA virions (V) for CpF97L^RT-^ (Fig. 5A, set 4) and in WT^RT-^ but at a significantly lower level (Fig. 5A, set 2). Because HBV cell cultures can generate significant amounts of naked capsids (Fig. 1 to 4) (15), an envelope minus (Env-) derivative of CpF97L was included as a control for the specificity of the HBsAg immunoprecipitation. Virions were not detected from Env- CpF97L (Fig. 5A, set 6) although RNA was accumulated in its intracellular capsids (Fig. 5A, set 5), signifying that the HBsAg immunoprecipitation was specific to extracellular enveloped virions and not to naked capsids.

In addition to the pgRNA, we detected faster-migrating RNA species in both intracellular capsids and virions. Because we were probing for the 5′-end of plus strand, this probe set would not detect the envelope sub-genomic RNAs. We speculated that the majority of the faster-migrating RNA would be spliced pgRNA as the HBV pgRNA generates spliced products, which contain the viral packaging signal ε and are packaged into capsids (25, 26). Indeed, in the poly A sample (Fig. 5A), we detected a band present in the WT^RT-^ and CpF97L^RT-^ lanes but absent in the IVT pgRNA lanes. We speculated that the band was Sp1 RNA as it has previously been characterized as the most abundant spliced variant (25).

To further characterize the RNA in capsids, we probed the Northern blots with oligonucleotides for HBV-plus nucleic acids, which lie in the major splice intron (ayw coordinates 2066 to 486), as this probe set should not detect the previously characterized, major spliced pgRNA species (26). With this probe set, as expected, we detected the full-length pgRNA band (Fig. 5B). We did not detect the putative Sp1 band, but two other faster-migrating bands (topmost label ***) were detected in the samples in addition to the pgRNA. These bands were also detected when the membrane was probed for the 3′-end of plus strand (nt 1326- to 1587-, data not shown). Based on the sizes of these RNAs and the speed of migration, we predict that these may be other HBV RNA, which has been identified in capsids and virions (27, 28).

To better understand the nature of the RNA species that were smaller than pgRNA, we made cDNA from the extracted intracellular capsid RNA and generated PCR products using primers upstream (ayw 1966+) and downstream (ayw 578-) of the splice intron. We detected three bands that were approximately 1,700, 600, and 200 base pairs. We then gel purified the excised bands and sequenced. Sanger sequencing indicated the presence of pgRNA (1,700 bp band), Sp1/6 (600 bp band), and Sp3 (200 bp band) virion RNA isolated from WT, WT^RT-^, and CpF97L^RT-^ cultures (data not shown). We also included companion samples in our analysis in which reverse transcriptase was absent from the cDNA reaction; no bands were detected in the PCR of these samples.

Together, these findings indicate that pgRNA, spliced pgRNA, and other HBV RNA are packaged into capsids and secreted in virions. Full-length pgRNA forms only a small percentage of the total encapsidated RNA.

### When reverse transcription is active, CpF97L secretes pgRNA virions in addition to dsDNA and ssDNA virions

In the preceding analyses in which we detected RNA virions for CpF97L, DNA synthesis was inhibited either with the Y63F mutation in polymerase or by the nucleoside analog entecavir. We wanted to ascertain if CpF97L secreted RNA virions when reverse transcription was active and DNA virions were being secreted. To do this, we extracted nucleic acids from intracellular capsids and purified virions, and treated the nucleic acids with RQ1 RNase-Free DNase. The resulting RNA was analyzed by Northern blotting and probed for nucleic acids of plus polarity as was performed in the analysis represented in Fig. 5.

**Fig 6:**
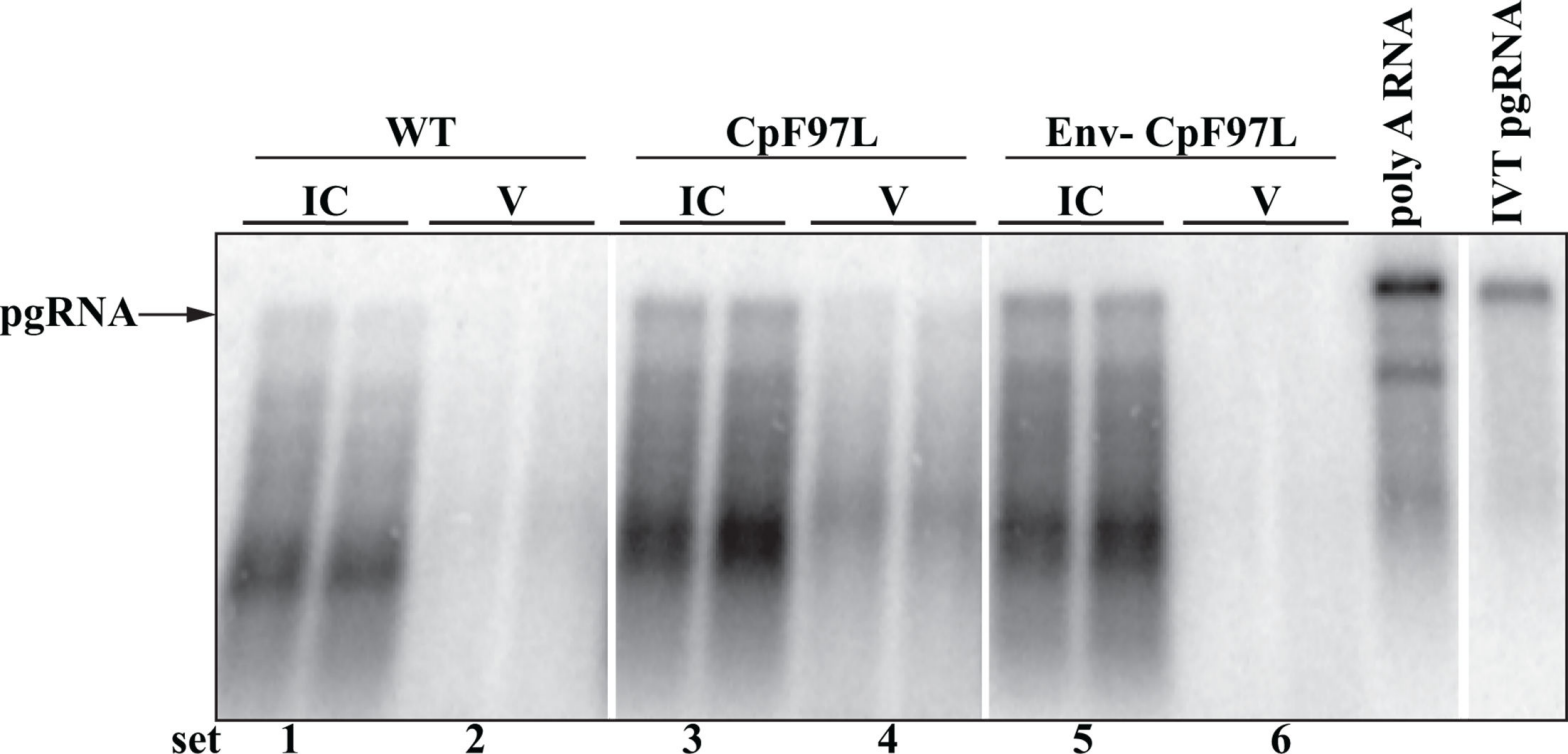
pgRNA virions are secreted when reverse transcription is active during the replication of Cp97L. Northern blotting of intracellular capsids (IC) and virion (V) RNA. IVT pgRNA, *in vitro* transcribed pgRNA; poly A RNA, total poly A RNA extracted from WT. IC samples represent the RNA from 1/10^th^ of the 60 mm plate; V samples represent the RNA from 1/3^rd^ of the total media harvested per transfected plate. The membrane was probed for the 5′-end of plus strand (ayw 2007- to 2498-). Representative data in Fig. 5 and 6 were analyzed together on the same membrane.

Similar to the results presented in Fig. 5, pgRNA and smaller HBV-derived RNAs were detected in capsids of WT and CpF97L intracellularly (Fig. 6, sets 1 and 3). RNA extracted from virions showed a similar profile (Fig. 6, sets 2 and 4) to intracellular capsid RNA. When reverse transcription was active, pgRNA was detected in virions from CpF97L. We did not detect a full-length pgRNA in WT virions. The profile of RNA detected in capsids mirrored the RNA detected when reverse transcription was inhibited (Fig. 5); pgRNA was present, but most of the RNA was shorter than full-length pgRNA.

### Proportions of dsDNA, ssDNA, and pgRNA in CpF97L virions

**Fig 7:**
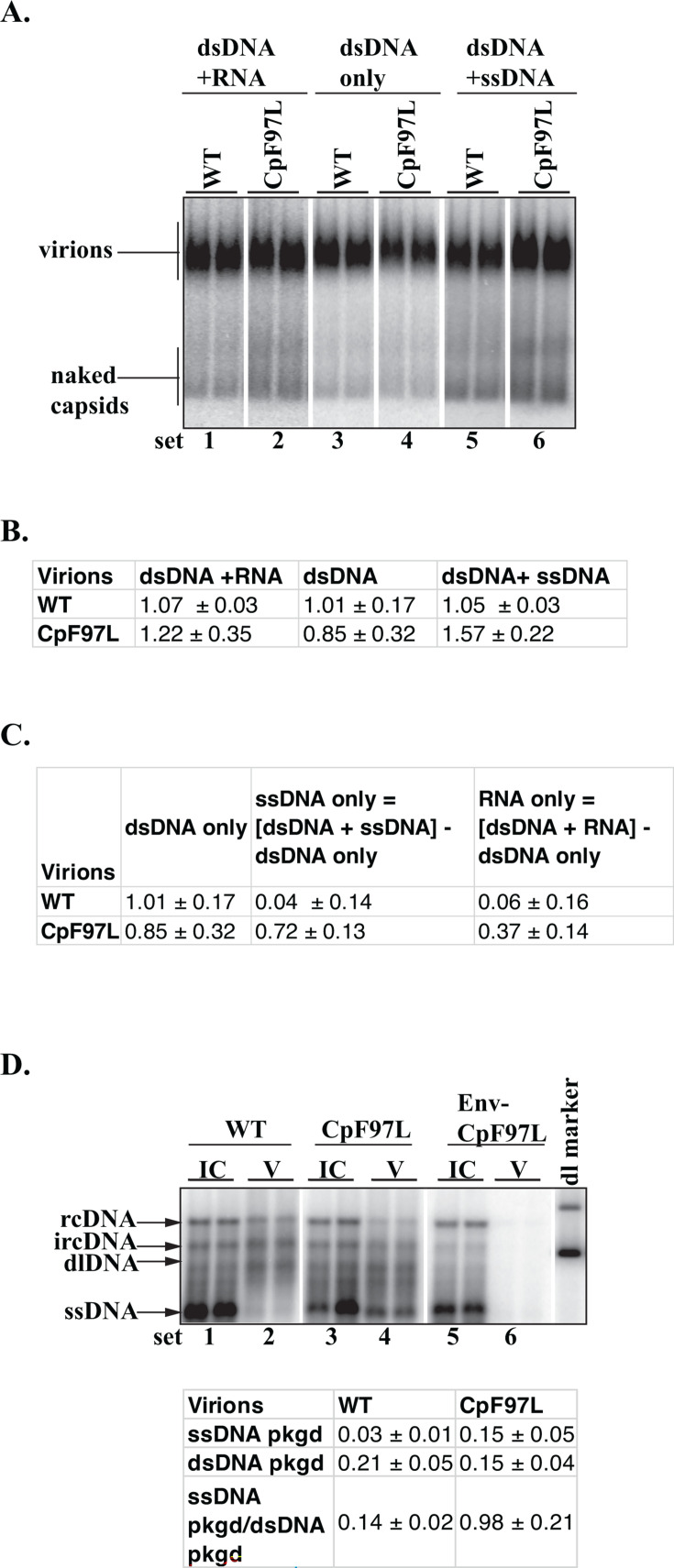
Proportions of pgRNA, ssDNA, and dsDNA virions secreted by WT and CpF97L. (**A**) The membrane resulting from the particle blotting of WT and CpF97L media, respectively, was probed sequentially; dsDNA + RNA, the 5′-end of plus strand; dsDNA, the 5′-end of plus-strand probing after RNase digest; dsDNA + ssDNA, minus-strand probing. WT and CpF97L samples were analyzed on the same membrane in each experiment. (**B**) Quantification of sequential probing of particle blot. Values (mean ± standard deviation) in the table were determined using the standard curve generated using WT media (as shown in Fig. 2). (**C**) Proportions of nucleic acids in WT and CpF97L virions derived by subtraction from sequential probing of particle blot (mean ± standard deviation). (D) Southern blot analysis of DNA isolated from WT or CpF97L. The ssDNA pkgd and dsDNA pkgd values were generated using the formula ($\frac{VssDNA}{ICssDNA+VssDNA}$) or ($\frac{VdsDNA}{ICdsDNA+VdsDNA}$), respectively, where dsDNA = (rcDNA + ircDNA + dlDNA). Data represent the mean ± standard deviation.

To determine the proportion of dsDNA, ssDNA, and pgRNA in CpF97L virions, we carried out particle blotting followed by sequential probing of the membrane. Briefly, the membrane was probed three times; first, for the 5′-end of plus strand to quantify dsDNA and RNA (Fig. 7A, sets 1 and 2). The same membrane was then treated with RNase A to remove RNA and then probed for the 5′-end of plus strand again to quantify the remaining nucleic acids as dsDNA (Fig. 7A, sets 3 and 4). We then probed the membrane for the 3′-end of full-length minus-strand DNA spanning ayw genome coordinates 1995+ to 2528+ to quantify ssDNA and the minus strand of dsDNA (Fig. 7A, sets 5 and 6). The plus- and minus-strand probe sets detected the same region of the genome (Fig. 1A). Between each probing experiment, membranes were stripped, exposed to a phosphor screen, and scanned to ensure the previous probe set had been completely removed.

We included a standard curve generated from WT media from which we expressed CpF97L nucleic acids relative to WT amounts (WT normalization). The standard curve was generated similarly to Fig. 2; 0.25×, 0.5×, 1×, and 2× volumes of media per WT plate were loaded onto the particle blot gel. 1× volume of media was loaded on the particle blot gel for CpF97L samples. The relative expression levels of CpF97L were determined by comparing the measured pixel volume to the WT standard curve. Compared to WT, CpF97L secreted 1.22 ± 0.35 (dsDNA + RNA) virions, 0.85 ± 0.32 dsDNA virions only, and 1.57 ± 0.22 (dsDNA + ssDNA) virions (Fig. 7B).

Because the same standard curve was used to derive each of the three virion quantifications (that is [dsDNA + RNA], dsDNA only, and [dsDNA + ssDNA] virions), we deduced the proportions of dsDNA, ssDNA, and pgRNA in CpF97L media by subtraction (Fig. 7C). The virions analyzed from WT cultures contained primarily dsDNA virions (Fig. 7C). For approximately every one dsDNA virion in WT, CpF97L secreted 0.85 ± 0.32 dsDNA virions, 0.72 ± 0.13 ssDNA virions, and 0.37 ± 0.14 RNA virions when reverse transcription was active.

To corroborate the ssDNA:dsDNA proportions detected by particle blotting, we extracted DNA from intracellular capsids and from virions isolated via IP with an HBsAg antibody. The DNA was analyzed by Southern blotting. We detected a ssDNA:dsDNA ratio of 0.98 ± 0.21 (Fig. 7D) for CpF97L. In our particle blotting analysis, we detected a ratio of approximately 1 (Fig. 7C), indicating congruence between the two methods.

Together, these results indicate that CpF97L is competent in secreting high levels of immature virions compared to WT.

## DISCUSSION

As has been reported historically, HBV CpF97L synthesizes high levels of ssDNA-containing virions in addition to dsDNA virions (20). In our work, we have found that CpF97L also secretes significant levels of pgRNA-containing virions and at a level much greater than WT HBV secretes pgRNA-containing virions. We determined that pgRNA virions were secreted from CpF97L cultures when we used the nucleoside analog entecavir (ETV) to block DNA synthesis via reverse transcription (Fig. 1). To corroborate our findings, reverse transcription was blocked by mutating the priming residue Y63F in polymerase (WT^RT-^ and CpF97L^RT-^). RNA-containing capsids accumulated in cells and were secreted at levels comparable to dsDNA in CpF97L^RT-^ (Fig. 1 and 2), as was observed when we blocked DNA synthesis by entecavir (Fig. 1). However, in WT^RT-^, pgRNA virions were inefficiently secreted, similar to what has been previously demonstrated by Ning et al. (15) and contrary to what was observed by Shen et al. (29).

We determined that RNA virion secretion was not a peculiarity of the cell line used for virus propagation but rather an intrinsic property of the CpF97L variant as RNA virions were secreted in CpF97L cultures in Huh7 as well as in HepG2 (Fig. 3), two of the most widely used cell lines in HBV research. To better understand the properties of immature secretion, we examined the secretion kinetics of dsDNA and RNA virions. We found that the secretion of dsDNA and pgRNA virions were similar. This conclusion is similar to the analysis examining ssDNA secretion in CpF97L (30). The similarity in the secretion kinetics of RNA and DNA virions (Fig. 4) suggests there may be other mechanisms distinct from capsid and/or genome maturity, such as the timing of envelope protein interactions, which may determine virion secretion kinetics (31). In this scenario, synthesis of dsDNA occurs quickly after pgRNA encapsidation, thus exerting little, if any, influence on the kinetics of virion envelopment and morphogenesis. Other factors such as capsid entry into the ESCRT pathway may have a larger impact on the kinetics of virion secretion.

In our analysis, HBV RNA found in virions was composed of pgRNA, spliced RNA, and other undefined HBV RNA species. Indeed, Shen et al. (29) identified RNA of heterogeneous lengths in virions from transfected cell cultures, cell lines stably expressing HBV, and from the serum of patients. Based on the probe sets used in our analyses, we cannot rule out the existence of other HBV RNA species in virions. For example, our probe set would not specifically detect HBV X mRNA, which others have identified in capsids and virions using 5′ RACE and Nanopore sequencing (27, 28). While we do not know why these less-than-full-length transcripts were packaged or secreted, there is some evidence to suggest that non-full-length HBV RNA may contribute to disease severity (32).

We detected pgRNA virions in CpF97L when reverse transcription was active (Fig. 6 and 7) in addition to ssDNA and dsDNA, indicating that immature virion secretion is a property of the CpF97L capsid. The Cp plays several vital roles in the successful replication of *Hepadnaviridae* viruses as it is involved in the encapsidation of the genome and reverse transcription (2, 3). In addition, it plays a critical role in virion morphogenesis and envelopment (2, 33) (Fig. 6 and 7). The so-called signal for envelopment and virion morphogenesis in HBV is unknown. It therefore remains to be determined what the relationship is between the amino acid 97 and the processes of envelopment and virion morphogenesis as the amino acid 97 sits in a hydrophobic pocket and not on the surface of the capsid (12, 34). Indeed, the change from the bulky phenylalanine to the less bulky leucine enhances the kinetics of assembly of the CpF97L capsid *in vitro* (34), although we (Fig. 4) and others (30) have shown that this does not change the kinetics of virion secretion. Furthermore, it remains to be determined if mutations in other viral proteins such as the envelope proteins can independently generate immature virions as has been observed in the Snow goose HBV (SGHBV), the other known Hepadnavirus that secretes ssDNA (33). In SGHBV, the capsid and envelope proteins are independently sufficient for the immature secretion phenotype.

As it is technically challenging, if not impossible, to cleanly separate and purify virions with distinct genomes (pgRNA vs ssDNA vs dsDNA), our understanding of the ability of pgRNA- or ssDNA-containing virions to initiate an infection remains limited. Our study demonstrates that the CpF97L variant could be used as a tool for generating high levels of RNA virions to help understand the role of pgRNA virions in the biology of HBV. For instance, it has been suggested (35) that a virus pool containing ssDNA virions may not be as infectious as one with predominantly dsDNA virions. It remains to be determined if RNA virions are as infectious as dsDNA virions, as to initiate an infection, the genome of a pgRNA-containing HBV virion would ultimately need to be converted into dsDNA and then cccDNA. Indeed, pgRNA introduced into the cytoplasm is able to generate cccDNA (36, 37). This would circumvent the need for dsDNA virions to propagate an infection, thus necessitating treatments that target all forms of HBV nucleic acids. Novel approaches to HBV treatment, such as combination therapies of reverse transcriptase inhibitors and use of capsid assembly modulators to effectively block cccDNA establishment, pgRNA encapsidation, DNA synthesis, and virion secretion, are needed (38). Further analyses are needed to elucidate the infectivity, or lack thereof, of HBV virions with pgRNA genomes.

The persistence of cccDNA in infected cells poses a major challenge in the search for an HBV cure. The proposal (28, 39, 40) to use RNA virions in patient serum as a surrogate marker for the cccDNA reservoir might be misleading as it is possible, if not likely, that other naturally occurring variants, in addition to CpF97L, secrete high levels of RNA virions. Caution should therefore be exercised in using serum levels of HBV RNA virions as an indicator for intrahepatic cccDNA levels. RNA virions in serum may not be an accurate representation of the cccDNA pool, and care should be taken in interpreting such data.

## MATERIALS AND METHODS

### Molecular clones and plasmids

All molecular clones of HBV were derived from the HBV genotype D, strain ayw (V01460 in GenBank). Nucleotide position one was designated as the C of the singular EcoR1 (GAATTC) restriction site within the genome in the plus polarity.

Plasmid TMA153 was used to engender wild-type HBV replication (41). Plasmid TMA153 uses the cytomegalovirus (CMV) immediate early promoter to express authentic pgRNA. Plasmid NU19 (WT^RT-^) is derived from TMA153 by substituting tyrosine with phenylalanine at codon 63 in polymerase (Y63F), which prevents the initiation of synthesis of minus-strand DNA. This results in the accumulation of pgRNA-containing capsids. Plasmid AKT9 contains the leucine codon, CTC, at position 97 of Cp in the TMA153 background and engenders the replication of HBV CpF97L. Plasmid AKT17 (CpF97L^RT-^) is the CpF97L equivalent of NU19; it does not undergo reverse transcription due to the Y63F substitution in P protein. CpF97L Env- does not express envelope proteins (42); S protein codon 1 has been changed from methionine to threonine (AUG to ACG), and codon 6 has been changed from serine to a stop codon (TCA to TAA). These nucleotide substitutions did not alter the amino acid sequence of the P protein.

### Cell cultures and transfections

All transfections were carried out using the human hepatoma cell lines Huh7 or HepG2, which were cultured in Dulbecco's modified Eagle medium/nutrient mixture F-12 (DMEM/F12) supplemented with 5% or 10% fetal bovine serum (FBS, SAFC Biosciences), respectively, at 37°C with 5% CO_2_. All media contained a final concentration of 100 U/mL penicillin streptomycin (Gibco). HepG2 cells were cultured on collagen-coated plates as previously described (43) to ensure a monolayer of adhered cells.

To engender HBV replication, cells were seeded in 60 mm culture plates 16 hours prior to transfection and were approximately 70% to 80% confluent at the time of transfection. Linear polyethyleneimine (PEI 25K, Polysciences) was used to transfect plasmid DNA into HepG2 or Huh7 cells at a mass ratio of PEI to DNA of 3:1 in Opti-MEM (43). An aliquot of 5 µg of HBV plasmid was transfected into cells along with 0.25 µg of a green fluorescent protein (GFP) expression plasmid and incubated for 6 hours. We estimated transfection efficiency by visualizing the fraction of GFP-positive cells by fluorescence microscopy. After 6 hours, the transfection media were removed, and the plates were washed with 1× phosphate-buffered saline(PBS). Fresh DMEM/F12 media supplemented with FBS were added. The media were replaced the next day, and the plates were incubated until harvest.

The medium that contained the virions was collected from each culture on days 3, 5, and 7 and was pooled together for subsequent analyses. Harvested media were clarified to remove cell debris by centrifuging at 2,500 rpm for 15 minutes at 4°C. Transfected cultures were harvested on day 7.

To inhibit HBV DNA synthesis, cells were grown in culture media supplemented with 1 µM (final) entecavir (22) (Selleckchem).

### Preparation of intracellular viral replicative intermediates and extracellular virions

Cell cultures were lysed with 500 µL of buffer comprised of 0.2% NP-40, 50 mM Tris, and 1 mM EDTA, pH 8.0, for 15 minutes at 37°C. The cell lysate was centrifuged to pellet the nuclei, which were then discarded. The supernatant was saved and considered to be a cytoplasmic lysate. To degrade nucleic acids not within a capsid, such as transfected plasmid DNA and unencapsidated pgRNA, the cytoplasmic lysate was adjusted to 2 mM CaCl_2_ and 44 U of micrococcal nuclease (New England Biolabs) then incubated at 37°C. After 2 hours, EDTA was added to a final concentration of 10 mM to chelate calcium ions and stop the micrococcal nuclease activity. The cytoplasmic lysate was used in one of two procedures: for nucleic acid extraction (DNA or RNA) or electrophoresed on a native agarose gel (particle gel).

The media that contained the virions were used either for native agarose gel electrophoresis (particle gel analysis) or for immunoprecipitation of virions with an HBsAg antibody followed by nucleic acid extraction.

For the particle gel analysis, the media were concentrated via polyethylene glycol 8000 (PEG, BD Sciences) precipitation. Typically, 2 mL of 30% PEG 8000 solution was added to 4 mL of media containing virions and incubated at 4°C overnight. The media were then centrifuged at 3,000 rpm for 15 minutes to pellet. The concentrated virion pellet was resuspended in 500 µL of Tris-buffered, serum-free media and adjusted to a final concentration of 1 mg/mL heparin for 10 minutes at room temperature to disassociate residual PEI-DNA complexes (Fig. S1) (44, 45). Then, the resuspended concentrated virion pellet was adjusted to 2 mM CaCl_2_ and 44 U micrococcal nuclease, and incubated at 37°C for 2 hours. The samples were then adjusted to 10 mM EDTA. The virions were precipitated a second time by addition of 250 µL of 30% PEG 8000 solution and incubation at 4°C overnight. The samples were centrifuged at 14,000 rpm for 5 minutes to pellet virions and then resuspended in 10 µL serum-free DMEM/F12 media buffered with Tris. Typically, the entire 10 µL solution was used in a single particle gel analysis.

To purify virions and remove naked capsids, immunoprecipitation with an HBsAg polyclonal rabbit antibody was carried out on clarified transfection media. Briefly, 4 mL of media was concentrated by PEG precipitation as described previously. The virion pellet was resuspended in 500 µL of Tris-buffered, serum-free media. The 10 µg of HBsAg polyclonal rabbit antibody (Fitzgerald, 20-HR20) (46) bound to 50 µL of protein A/G magnetic beads (Pureproteome, Millipore) was added to the concentrated media and incubated overnight at 4°C. The next day, a magnetic rack was used to separate the IP beads from the supernatant. The beads were washed 3× with PBS-T and then resuspended in 500 µL of Tris-buffered, serum-free media for nucleic acid extraction.

### Nucleic acid (DNA and RNA) extraction

Nucleic acids were extracted from cytoplasmic lysates that contained capsids and from virions isolated from media via immunoprecipitation using a similar procedure. First, all samples were treated with micrococcal nuclease to degrade nucleic acids not within each respective particle type. Next, the samples were adjusted to 10 mM EDTA and 0.4% SDS, and treated with 400 µg/mL Pronase (Roche) at 37°C for 2 hours to digest Cp and P proteins. Next, the samples were extracted with a 1:1 mixture of phenol–chloroform, followed by chloroform. Finally, the nucleic acids were precipitated from each sample with ethanol.

For the analysis of DNA only, RNA was removed from the sample by treatment with RNase A. Reciprocally, for the analysis of RNA only, DNA was removed by treating the sample with DNase (RQ1 RNase-Free DNase, Promega).

### Native agarose gel particle blotting (particle gel blotting)

Intracellular cytoplasmic capsids and media-derived virions were electrophoresed through a 1% agarose-Tris-acetate-EDTA(TAE) gel and then transferred, via capillary action, with 1× TNE buffer (10 mM Tris, 150 mM NaCl, and 1 mM EDTA, pH 8) to a positively charged nylon (*N*+) membrane (Amersham Hybond or Invitrogen Brightstar) overnight at room temperature. After the transfer, the membrane was washed once in water for 5 minutes and then allowed to air dry. To denature the capsids and/or virions and their nucleic acid contents, the membrane was incubated for 10 seconds in a solution of 0.2 N NaOH/1.5 M NaCl. The membrane was then neutralized by incubating in a solution of 1 M Tris-HCl/1.5 M NaCl, pH 4.5, for 5 minutes. The membrane was washed once with water for 5 minutes and allowed to air dry. The nucleic acids were cross-linked to the nylon membrane by applying 120,000 μJ/cm^2^ of UV photons at a wavelength of 254 nm.

### Southern blotting

DNA isolated from intracellular capsids or extracellular virions was electrophoresed in a 1.25% agarose-Tris-borate-EDTA(TBE) gel. The gel was then denatured in 1 M NaOH and 1.5 M NaCl, and neutralized in 1 M Tris HCl, 1.5 M NaCl, pH 7.4. The gel was transferred by capillary action overnight in 10× saline sodium citrate (SSC), pH 7, to a nylon membrane. Then, the membrane was washed in water, allowed to air dry at room temperature, and cross-linked by UV as described above.

### Northern blotting

Northern blotting was performed as previously described (47). RNA samples were resuspended in a formaldehyde-formamide solution and denatured at 65°C for 5 minutes. The samples were immediately chilled in iced water (4°C) and loaded on a 1.5% 3-(N-morpholino)propanesulfonic acid (MOPS) agarose gel with formaldehyde. The gel was electrophoresed in 1× MOPS buffer. After electrophoresis, the transfer was set up similarly to the Southern blot; the gel was transferred by capillary action in 10× SSC, pH 7, to a nylon membrane overnight. The membrane was cross-linked by UV immediately after the transfer.

### Membrane probing and quantification

All membranes were probed with sets of oligonucleotides end-labeled with γ-^32^P ATP (Perkin Elmer/Revvity) as described by Lewellyn and Loeb (23). See Table 1 and Fig. 1 for the location of the different oligonucleotide probes. Briefly, the membranes were pre-incubated in Church hybridization buffer (0.25 M Na_2_HPO_4_, 5 mM EDTA, 1% bovine serum albumin, 7% SDS, pH 7.2) at 45°C for at least 15 minutes prior to the addition of labeled oligonucleotide probes, followed by incubation at 45°C overnight. The next day, the solution containing the hybridization probes was removed, and membranes were washed at least five times with Church Wash Solution (1% SDS, 20 mM Na_2_HPO_4_, 1 mM EDTA, pH 7.2) at room temperature and then air dried. The membranes were exposed to a phosphoscreen (Molecular Dynamics) and visualized on the Typhoon TRIO Variable Mode Imager (GE Sciences). Nucleic acid bands were quantified using the ImageQuant 5.2 software.

**TABLE 1:** Summary of oligonucleotide probes

Probe	ayw coordinates	Number of oligos	Total nucleotides covered (bp)
5′-end of plus strand	2007- to 2498-	20	~400
3′-end of minus strand	1995+ to 2528+	20	~400
Major splice intron on plus strand	2499- to 3119-	6	126

All Southern blots included linearized plasmid DNA of known mass to generate standard curves for quantifying the mass of each sample. In addition, the linearized plasmid DNA contained a fragment of 3,182 bp to allow the identification of the duplex-linear, replicative intermediated DNA species (dlDNA).

To quantify the particle blots, we generated standard curves from WT intracellular capsids or virions (Fig. 2) and expressed the levels of the HBV nucleic acids relative to WT. Briefly, amounts corresponding to 0.25×, 0.5×, 1×, and 2× volumes of media were loaded on each gel. An equation of a straight line was determined by plotting the volume of media loaded against the pixel volume measured. The level of each sample was determined by comparing the pixel volumes to the WT standard curve and was expressed as a fraction of the WT level from that transfection.

### Removal of probe sets from previously probed membranes

To remove the hybridization probes from previously probed membranes, the membranes were incubated in a solution of TE buffer (10 mM Tris, 1 mM EDTA, pH 8) and 1% SDS at 80°C for 30 minutes. The membranes were then washed twice with nuclease-free water at 80°C, air dried, and then exposed to a phosphor screen overnight to ascertain that the hybridization probes were successfully removed.

### Removal of RNA from previously probed particle gel membranes

To detect only the plus-strand DNA and not the RNA, we removed the RNA from the previously probed membranes and then re-probed the membranes afterward. First, the hybridization probes were removed from the membranes as described above. The membranes were then incubated with 0.01 mg/mL RNase A in buffer (0.3 M NaCl, 50 mM Tris, pH 8, 1 mM EDTA) for 15 minutes at 37°C to digest the RNA on the membranes. The membranes were then washed twice with 2× SSC at 25°C and rinsed once with nuclease-free water. We then probed again for plus strand to detect only the DNA.

## Data Availability

Any data supporting the findings of this study are available upon request from the corresponding author.

## SUPPLEMENTAL MATERIAL

Fig. S1 jvi.00100-25-s0001.pdf Representative image showing a combination of heparin and micrococcal nuclease treatment is effective in degrading DNA-PEI complexes.

ASM does not own the copyrights to Supplemental Material that may be linked to, or accessed through, an article. The authors have granted ASM a non-exclusive, world-wide license to publish the Supplemental Material files. Please contact the corresponding author directly for reuse.
