# Supplementary material for: The HBV variant CpF97L supports the secretion of pgRNA-containing virions at a level much greater than WT HBV
Source: J Virol. 2025 Apr 15;99(5):e00100-25. doi: 10.1128/jvi.00100-25 (PMC12090816; doi:10.1128/jvi.00100-25)
Supplement: Fig. S1 — Representative image showing a combination of heparin and micrococcal nuclease treatment is effective in degrading DNA-PEI complexes. [file jvi.00100-25-s0001.pdf]

The HBV variant, CpF97L, supports the secretion of pgRNA-containing virions at a level much greater than WT HBV.

Abena Adomah Kissi-Twum,[a,b] , Karolyn Pionek,[a*] and Daniel D. Loeb[a]#

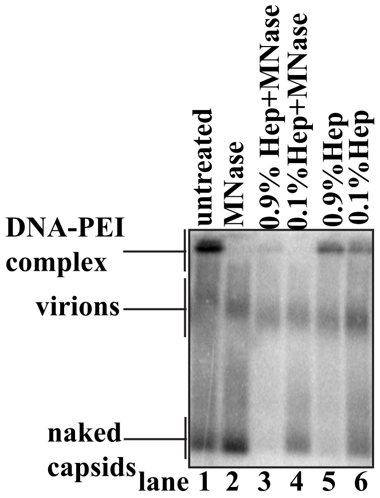

Figure S1: Representative image showing a combination of heparin and micrococcal nuclease treatment is effective in degrading DNA-PEI complexes. Media harvested from Huh7 cells transfected with WT virus was analyzed by particle blot. Five different treatments were tested (lanes 2-6). As indicated in the Materials and Methods the treatment in lane 4 was used throughout this paper.
